# Seasonality impacts collective movements in a wild group-living bird

**DOI:** 10.1186/s40462-021-00271-9

**Published:** 2021-07-08

**Authors:** Danai Papageorgiou, David Rozen-Rechels, Brendah Nyaguthii, Damien R. Farine

**Affiliations:** 1 Department of Collective Behavior, Max Planck Institute of Animal Behavior, Universitätsstraße 10, 78457 Constance, Germany; 2 Department of Biology, University of Konstanz, Universitätsstraße 10, 78457 Constance, Germany; 3 Center for the Advanced Study of Collective Behaviour, University of Konstanz, Universitätsstraße 10, 78457 Constance, Germany; 4 Department of Evolutionary Biology and Environmental Studies, University of Zurich, Winterthurerstrasse 190, 8057 Zurich, Switzerland; 5 Kenya Wildlife Service, P.O. Box 40241-001000, Nairobi, Kenya; 6 School of Natural Resource Management, Department of Wildlife, University of Eldoret, Eldoret, 1125-30100 Kenya; 7 Mpala Research Center, P.O. Box 92, Nanyuki, 10400 Kenya; 8 Department of Ornithology, National Museums of Kenya, P.O. Box 40658-001000, Nairobi, Kenya

**Keywords:** Avian ecology, Climate change, Collective movement, Drought, Home-range, Movement ecology, Seasonality, Space-use

## Abstract

**Background:**

A challenge faced by animals living in groups with stable long-term membership is to effectively coordinate their actions and maintain cohesion. However, as seasonal conditions alter the distribution of resources across a landscape, they can change the priority of group members and require groups to adapt and respond collectively across changing contexts. Little is known about how stable group-living animals collectively modify their movement behaviour in response to environment changes, such as those induced by seasonality. Further, it remains unclear how environment-induced changes in group-level movement behaviours might scale up to affect population-level properties, such as a population’s footprint.

**Methods:**

Here we studied the collective movement of each distinct social group in a population of vulturine guineafowl (*Acryllium vulturinum*), a largely terrestrial and non-territorial bird. We used high-resolution GPS tracking of group members over 22 months, combined with continuous time movement models, to capture how and where groups moved under varying conditions, driven by seasonality and drought.

**Results:**

Groups used larger areas, travelled longer distances, and moved to new places more often during drier seasons, causing a three-fold increase in the area used at the population level when conditions turned to drought. By contrast, groups used smaller areas with more regular movements during wetter seasons.

**Conclusions:**

The consistent changes in collective outcomes we observed in response to different environments raise questions about the role of collective behaviour in facilitating, or impeding, the capacity for individuals to respond to novel environmental conditions. As droughts will be occurring more often under climate change, some group living animals may have to respond to them by expressing dramatic shifts in their regular movement patterns. These shifts can have consequences on their ranging behaviours that can scale up to alter the footprints of animal populations.

**Supplementary Information:**

The online version contains supplementary material available at 10.1186/s40462-021-00271-9.

## Background

Ecological processes shape movement decisions of individuals. By changing the abundance and distribution of resources, inter-seasonal meteorological variability affects individual behaviour and physiology [1–3]. In environments with evident seasonality, plasticity in social (e.g. changing social organisation [4]) and movement decisions (e.g. foraging trips, migration) could buffer the effects of environmental variations on physiology [5, 6]. For animals that live in social groups with stable membership—where individuals remain together throughout the year—seasonal conditions can add the additional challenge of requiring group members to achieve different collective actions as conditions change. For example, African elephants (*Loxodonta africana*) alter their normal movements during drought by navigating in new areas to find resources, despite these being previously unknown to most group members [7]. Such outcomes can be the result of group properties that emerge from individual actions. In colonial ants, outgoing foragers available to leave the nest start their next foraging trip based on the rate of contacts with foragers returning with food, and thus changing food availability determines collective foraging effort [8]. It has been proposed that the mechanisms that underpin how groups make collective decisions should reflect the environmental conditions that they experience [9]. But what happens when groups of non-migratory species with stable group membership experience large-scale changes to their environments—such as the dramatic changes from dry to wet seasons? To date, the emerging patterns of collective behaviour have predominantly been studied in lab environments [10] or for short periods of time in the wild [11], with the latter often relying on low-resolution data collection methods. As a result, we currently lack insight into the range of environmental conditions that social groups face in natural, seasonal environments, and therefore the types of actions that groups must achieve in order to survive as their environments change and how these changes scale up to determine the landscape-level footprint of animal populations.

Living in a group requires balancing costs, such as within-group competition, and benefits, such as those gained from safety in numbers or information sharing [12]. To maintain group cohesion, individuals may sometimes have to prioritize following their group rather than fulfilling their own needs. In seasonal environments, the balance of costs and benefits could shift, inducing a modulation of social behaviour. In some species, such modulations correspond to a complete restructuring of the social system (i.e. species that switch seasonally from being territorial to group-living [4]) or to changes in group structures (i.e. fission-fusion dynamics [13, 14];). However, in many species, individuals live in the same group for extended periods of time—in extreme cases for their entire lives [15]—meaning that changes in the costs borne by the interaction of environmental fluctuation and group-living should be modulated through behavioural changes, including those expressed at the group level. For example, if individuals have to adjust their movement patterns under different environmental conditions to satisfy their nutritional needs [16], then groups must find solutions to allow these changes to occur, while maintaining cohesion, so that the nutritional needs of all group members can be satisfied. Gaining detailed information on changes in behaviour by social groups across seasonal conditions and environmental extremes is critical for understanding and making predictions about how social species might adapt in an era of global environmental change [17].

Seasonal environments often result in conditions ranging from high resource availability in one season to low resource availability in another [18]. These extremes can correspond to spring (temperate species) or rainy seasons (tropical species) versus winter (temperate species) or dry seasons (tropical species), respectively. Seasonal changes are often associated with different priorities for group members. For example, individuals generally invest in reproduction during periods of high resource availability [19], whereas periods of low resource availability require investing in finding sufficient food, water or shelter to survive [7, 20]. For animals that remain in groups during dry seasons, one prediction is that groups should move more and range over larger areas relative to non-group-living individuals, as optimal foraging theory predicts that groups deplete resources more efficiently than individual animals in the same environment, and thereby spend a higher proportion of their time moving between patches [21]. Such outcomes—for example travelling farther or having to move more often to new areas—should become more extreme as conditions become less favourable, such as during severe droughts when resources are more unpredictably distributed [22]. Given increasing weather unpredictability, and more numerous periods of more intense and longer droughts, under climate change [23], a key question is whether animals living in stable groups can express sufficiently flexible group-level behaviours to allow them to survive such extreme conditions. Collective behaviour may provide solutions, such as improving the ability for individuals to find resources [24] through collective intelligence [25]. However, it may also induce challenges if it limits the options available to individuals that live in cohesive groups relative to individuals that live in more open societies. For example a small patch might provide sufficient resources for an individual or a small group, but not for a larger group [26]. Addressing this gap requires a better understanding of how group behaviours change over the spectrum of conditions that each group is likely to experience.

Groups might respond to seasonal conditions in a number of ways. One way is to increase their home range. A home range represents the area within which a group moves to find resources essential for the reproduction and survival of its members [27]. In some species, groups can express flexible home ranges, allowing them to increase their home range size when resources become scarcer (territorial species might be more limited in their ability to exhibit such plasticity in home range use). For example, baboon groups travel less in years with increased rainfall [16]. Larger home ranges can be achieved in one of two non-exclusive ways. First, groups can increase their daily travel distance, how far they travel each day, to cover larger areas [28]. Second, they can reduce the day-to-day fidelity in their space use by travelling to different parts of their home-range in different days [29]. In extreme cases, groups can shift their home range completely across different seasons to range in areas with more resources. Just as the behaviour of individuals drives emergent properties at the level of the group, such changes in group-level outcomes could then scale-up to affect the emergent properties of populations. It could thus be possible that some group-living species may also be impacted by limitations in how groups can modulate their behaviour under different conditions or by the space available for them to expand their home range during seasons with a scarcity of resources. However, addressing such questions has been challenging [30, 31] as it requires detailed tracking of replicated groups over long time periods to capture sufficient information and inform data-intensive metrics, such as home-range size and daily travel distance, and how these change over changing environmental conditions (i.e. seasons).

Here, we examined the effect of seasonality on space use in wild groups of vulturine guineafowl (*Acryllium vulturinum*). Vulturine guineafowl live in a multilevel society that consists of large and cohesive groups with stable membership [32]. These groups associate preferentially in space and time with specific other groups, without presenting signs of territoriality [32]. We used data from high-resolution solar-powered GPS tags fitted to 57 individuals, distributed across each of the distinct groups living in our population over 22 months. We partitioned these data into 11 two-month-long study periods, with each period having distinct seasonal conditions that are typical of our study area [33]. During our study, we captured two extreme seasonal conditions, extreme drought and very wet seasons. From these data, we calculated home range size (Question 1), daily travel distance (Question 2), daily range fidelity (Question 3) and seasonal range fidelity (Question 4). We then tested how these patterns impacted the population-level footprint (Question 5).

Vulturine guineafowl are not bounded by territorial boundaries, allowing multiple groups to travel to the same resources [32]. We therefore expected that groups should utilize larger areas, travel longer distances, and explore new areas in the search for resources in study periods with drier conditions relative to those with wetter conditions. Because the distribution of these resources might be predicted by environmental conditions, we also predicted that the home range similarity across study periods should be higher in seasons with more similar conditions (e.g. across two dry seasons relative to a wet and dry season). Lastly, because of the reduced density of resources as conditions become drier, we expected our study population to expand to occupy a much wider area during drier periods relative to wetter periods.

## Methods

### Study site and study species

We studied a population of vulturine guineafowl that resides in a savannah-woodland ecosystem of approximately 12 km^2^ in the southern part of the Mpala Research Conservancy (MRC) in Laikipia, Kenya. The geographical features immediately surrounding the research area function as boundaries as they consist of habitat that is unsuitable for vulturine guineafowl. That implies that our study population is relatively self-contained, with non-study groups mostly occurring along the southern and the eastern boundary of the study area.

Vulturine guineafowls are large (~ 1.5 kg), predominantly terrestrial, and highly gregarious birds that live in groups with largely stable membership, ranging in size from 13 to 65 adults and moving cohesively (see details in Papageorgiou et al. [32]). Groups have a very steep dominance hierarchy, with males dominant over females, but use a shared decision-making system, meaning that any group member can initiate movement towards their preferred direction (albeit this is rarely successful for juveniles less than 10 months old) [34]. Previous work on decision-making in vulturine guineafowl social groups found that leadership (a within-group process) is impacted by the distribution of resources, with more clumped resources driving subordinates to influence where the group moves [34].

Despite their stable group membership, vulturine guineafowl are not territorial; they roost communally in trees that can contain several hundred birds and regularly encounter other groups during the day, at which times between-groups agonistic interactions are rarely observed [32]. Vulturine guineafowl are therefore ideal for studying group movements because groups can move freely, unbounded by territorial boundaries, but maintain a relatively consistent home range over the course of each season [26, 32]. Birds breed during wet seasons in which sufficient rainfall has occurred, with a breeding season usually lasting around 2 months. In such periods, multiple breeding pairs split from their group to breed. Males return to their group when the female starts incubating her eggs and females return to their group when their chicks hatch. Based on our field observations, birds are prone to ambush by carnivorous mammals, such as jackals or leopards, when moving through dense vegetation, and are an important prey item for raptors, such as martial eagles [35], to which they are susceptible to predation when in open areas.

### Movement data collection

The large body size (~ 1.3–1.9 kg) of adult vulturine guineafowl allowed us to fit individuals with high-resolution solar-powered GPS tags to collect both fine-scale data on their movement and long-term data on their ranging behaviour. We fitted GPS tags (15 g Bird Solar, e-obs Digital Telemetry, Grünwald, Germany) set to collect daily movement data to between one and five individuals in each of the groups in our study area (57 individuals in total, see below for details on defining group membership). GPS tags, together with the harnesses and the platform used to elevate the tag and thus prevent the solar panel from being covered by feathers, weighed less than 2% of a small bird’s weight, which aligns with animal welfare guidelines. GPS tags collected data from 06:00 to 19:00 (representing dawn until dusk), frequently recording at high resolution (1 Hz) (~ 25% of the time, when the batteries were fully charged), and collecting a burst of 10 points every 5 min at other times (i.e. when the batteries weren’t fully charged). For more details on trapping the birds, tag deployment, programming, and data storage, see Papageorgiou et al. [32]. For the present study, we used data collected daily from the 1st of May 2018 until the 21st of April 2020 (22 months in total).

### Study periods

As our field site is on the equator, seasonality is strongly characterised by two wet seasons (high rainfall) interspersed by dry seasons (low rainfall) [32, 33]. Rainfall determines grass cover as well as the availability of insects, which characterise the diet of vulturine guineafowl alongside grass root buds, seeds, and other small invertebrates. Rainfall can vary greatly between years but typically there is a short rainy season (October–November), followed by longer period of dry weather (December–February), which some years can be more extended into April causing drought. Long rains take place from as early as March and can last through to June. After the end of each rainy season there is often an intermediate season during which conditions remain green with occasional rainfalls. If rainfall remains low for a couple of months, conditions become very dry (e.g. trees lose their leaves, there is no green vegetation).

**Fig. 1:**
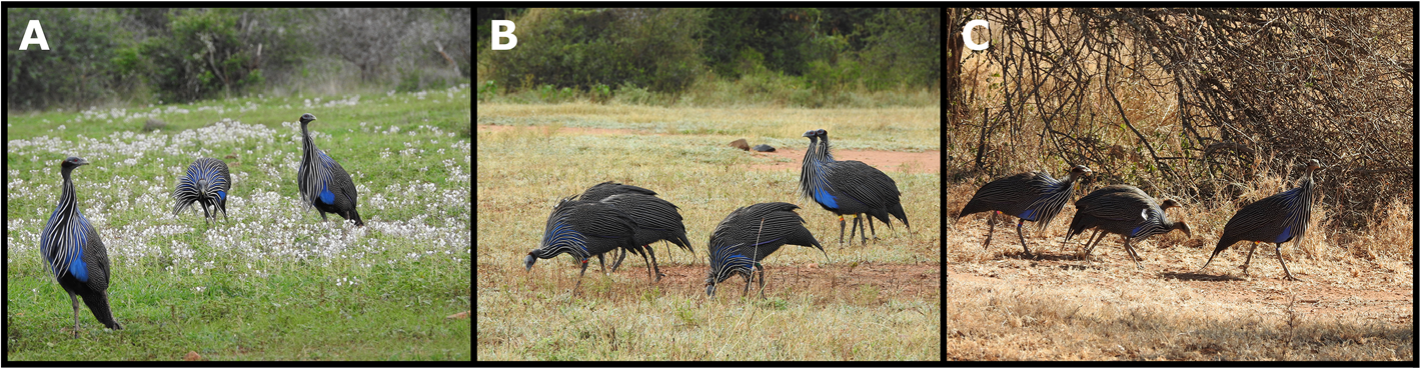
Classification of study periods. Study periods were classified as **A** wet, **B** intermediate, or **C** dry, with the classification based on rainfall data from a local weather station and NDVI [36] as a measure of the greenness of the grass throughout each study period

To make our data comparable across our study, we partitioned the 22 months dataset into 11 replicated two-month-long study periods. This ensured that we had similar amounts of data for each seasonal period (thus not biasing some seasons as, for example, having more data would result in larger home ranges). We a priori defined the start and end of each two-months span to correspond with distinct seasonal changes in seasonal conditions, such as the onset of rain, based on our observations of the habitat and weather in the field (see Fig. 1). We then used data from a weather station at the Mpala Research Centre [37] and Normalized Difference Vegetation Index (NDVI) from satellite imagery [36] to classify each of our two-month study periods into one of three seasonal categories: wet, intermediate or dry (see Supplementary Table 1 and Supplementary Figure 1 for details). We also investigated measuring NDVI on glades, as glades are the typical foraging areas for our study species, but found that NDVI measured in the entire landscape and in the glades are highly correlated (NDVI_glades_ = 1.15 × NDVI_landscape_ − 0.07, Pearson’s R = 0.98).

### Characterising group membership

Because multiple individuals from the same group were fitted with GPS tags, we identified which birds were in the same group in each of the study periods. We estimated group membership in each study period based on the proximity of each dyad of GPS birds in our study population. Proximity networks were based on estimating the proportion of simultaneous detections between two individuals being in close proximity, which we had previously defined (based on whole-group tracking) to be 28.67 m [32]. We thresholded these networks to keep only dyads that were observed together more than the 20% of the time, to avoid potentially categorizing birds from different groups that roost and forage together in the mornings and evenings, but move separately during the day, into the same group. We then ran the fastgreedy community algorithm, from the R package igraph [38], on each of the proximity networks, to identify which clusters of connected birds formed part of the same group. The fastgreedy community algorithm detects communities in networks (sets of nodes that are more interconnected to each other than to other sets of nodes) by directly optimizing a modularity score [39]. In previous work [32] we demonstrated a strong match between GPS-based proximity networks and networks collected from census observations of co-occurrences among colour-banded individuals (Mantel correlation test between two-month census- and GPS- derived networks: r > 0 .81, for more detailed results see Data S1D in Papageorgiou et al. 2019 [32]). However, because during some seasons we could not observe some groups, we opted to use GPS-based networks for the current study.

Because group memberships should be auto-correlated across subsequent periods, we next identified the carry-over of group membership from one season to the next. For this, we used the MajorTrack python library [40], which identifies the links between communities across time periods. Because of the long-term stability of groups in our system, we used a long history (8 seasons) over which to estimate carry-over of group membership identity, although the results were not sensitive to this choice. The algorithm identified 27 distinct group identities in our population. Because sometimes groups occasionally aggregated into one larger group or split into multiple smaller groups from one period to another, not all groups spanned the entire study period. Further, because on some occasions all the GPS tags in one group were removed and replaced with tags on new group members, we manually connected some otherwise disconnected communities. The number of groups we tracked varied from 12 to 26 per period, and increased over time, which was partly due to the increased number of groups tracked as our study progressed and also because of several breeding events causing group splits.

### GPS data pre-processing

In the current study, we used data points collected on the tenth second of every fifth minute. Given that the GPS tags mostly operate on collecting ten positions starting in the first few seconds after every 5 min, using the tenth fix ensured we allowed the tag time to switch on and improve its accuracy as it detects more satellites, while also ensuring we collected data at precisely the same time for each bird. When tags collected high-resolution (1 Hz) data, we also subset to keep only the fix 10 s after every fifth minute. Our sampling therefore allowed the data points from all GPS-tagged birds to be synchronised in time.

### Movement analysis

We used the GPS data from each individual in each study period to quantify key aspects of group movement characteristics. Specifically, we calculated (1) the core home range (50% auto-correlated kernel density estimate), (2) the daily travel distance, (3) the day-to-day site fidelity and (4) season-to-season range fidelity. Finally, we (5) quantified how the footprint of our population changed across seasons. For (1) and (2) we used data from all individuals of all groups. However, for computational limitations, we selected one individual per group for questions (3–5).

To estimate the core home range size (1), we fitted a continuous time movement model (ctmm) to each individual’s GPS data for each study period. These models follow a continuous-time stochastic process from which the maximum likelihood Gaussian home range area can be extracted after the best fitted model is selected based on AIC, these areas are termed as AKDEs (auto-correlated kernel density estimations). Because ctmms model the actual movement, they better predict home ranges than classical approaches, such as minimum convex hulls, that are typically prone to being highly influenced by just a few data points [30, 41]. We used the maximum likelihood home ranges to determine the area in which each bird was located 50% of the time. This procedure was done using the “ctmm” R package [42, 43]. We selected a 50% home range to avoid over-estimating the home range sizes when groups move across larger areas (see Supplementary Figure 2) but we also provide the analysis of the 95% AKDE polygons in the Supplementary Results.

To calculate the daily travel distance (2) for each individual in each study period, we applied the function “speed” from the ctmm R package to the five-minute data. This function calculates the mean distance covered per unit time, which generates a mean daily travel distance across the study period when setting the unit of time to 1 day.

For day-to-day site fidelity (3), which reveals the extent to which animals explored new areas, we first fitted a ctmm to the daily five-minute data from one representative for each group. We then calculated the overlap of the daily auto-correlated kernel density estimation for daily calculated 50% AKDE home ranges using the “overlap” function of the “ctmm” R package [44]. Finally we calculated the mean of the values representing the overlap across each consecutive set of 3 days to generate a measure of fidelity in each study period for each group.

To calculate the season-to-season overlap (4), we used the “overlap” function of the “ctmm” R package to estimate the 50% AKDE for each individual representing each group across each pairs of study periods.

To estimate the total area used (5) by our study population in each different season, we pooled hourly GPS data of each study group in each season. Because these data could not be used to fit ctmms, we calculated a core area (50%) and a wider area (95%) using a Kernel Density Estimation (KDE). We did this calculation using the package adehabitatHR [45] and the default ‘href’ kernel smoothing factor.

### Statistical analysis

We fitted linear mixed models (LMMs) [46] in R version 3.5.1 [47] to test if season type (wet, intermediate, dry) predicts home range size, daily travel distance, day-to-day site fidelity and seasonal range fidelity. The fixed effects of the models for (1), (2) and (3) were the season type and the number of days for which we had tracking data available (range: 41–61 days per study period, we scaled this variable to be considered in the model). To account for repeated observations of the same groups across study periods, we fitted group identifier as random effect. For questions (1–3) the reference level of season type was set to dry seasons and for question (4) the reference level was the overlap between two dry seasons. We finally used the lmerTest package to run Type III analysis of variance on the calculated LMM, extracting *P*-values for F-tests and t-statistics following the Satterthwaite’s approximation method [48]. In the main text below we provide the results of the F-tests and in the supplementary material tables we provide the summary tables for each model.

## Results

**Fig. 2:**
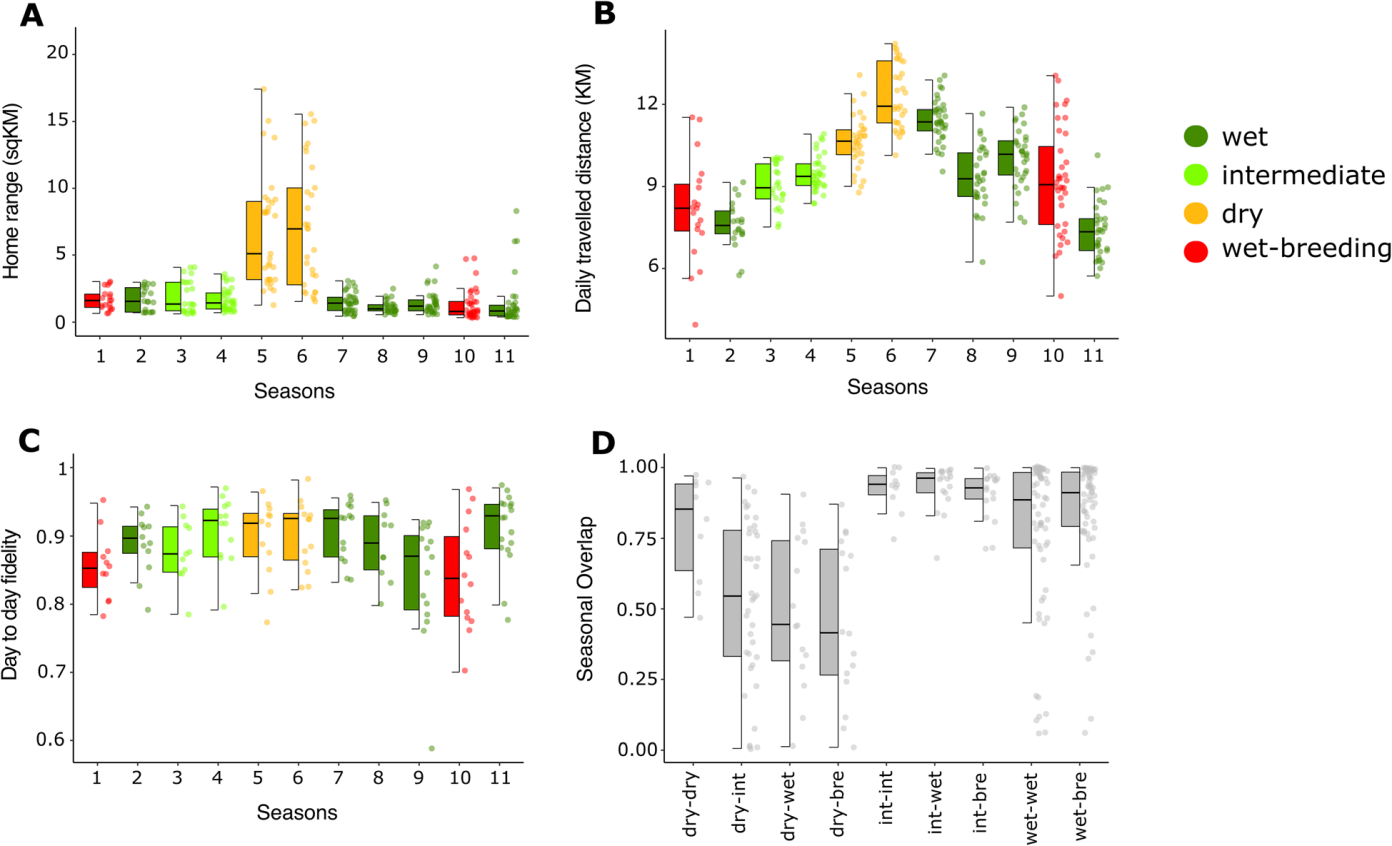
Group movement characteristics change in response to seasonality. We included 11 consequent two-month study periods in our analysis that were classified as wet, intermediate, and dry. The study population bred in wet seasons 1 and 10. Panels show for each study group **A** 50% core home range in square kilometres size per season, **B** daily travelled distance in kilometres per season, **C** day-to-day space use overlap per season and **D** seasonal overlap per season type

In total, our dataset comprised of 161,258,429 GPS points. These data revealed that the 50% core AKDE home-range size was around 85% smaller in wet and intermediate seasons than in dry seasons (Fig. 2A, Model summary presented in Supplementary Table 2, *F*_3_,_318.6_ *=* 41. 916*, p* < 0.001). We found similar patterns for the 95% AKDEs (Supplementary Table 3). Correspondingly, we found seasonal effects on daily distance travelled (*F*_3,314.59_ *=* 42.021, *p* < 0.001), with groups having around 23% shorter daily travel distances in intermediate and wet seasons compared to dry seasons (Fig. 2B, Model summary presented in Supplementary Table 4). We also found a significant effect of season on day-to-day site fidelity (*F*_3,120.75_ *=* 4.231*, p* = 0.007), with greater day-to-day fidelity in dry seasons than during when the birds were breeding (seasons 1 and 10), although there were no significant differences between dry seasons and wet or intermediate seasons during which breeding did not occur (Fig. 2C, Model summary presented in Supplementary Table 5). Further, we found significant differences in range overlap between different season types (*F*_8,236.82_ = 16.699, *p* < 0.001), with the overlap in range between dry seasons being more than twice the overlap in home range between wet and dry seasons (Fig. 2D, Model summary presented in Supplementary Table 6). Finally, our population-level data revealed that the overall population footprint—the area used by all groups combined—expanded during dry seasons to more than three times the area used in intermediate or wet seasons (Supplementary Table 1).

## Discussion

**Fig. 3:**
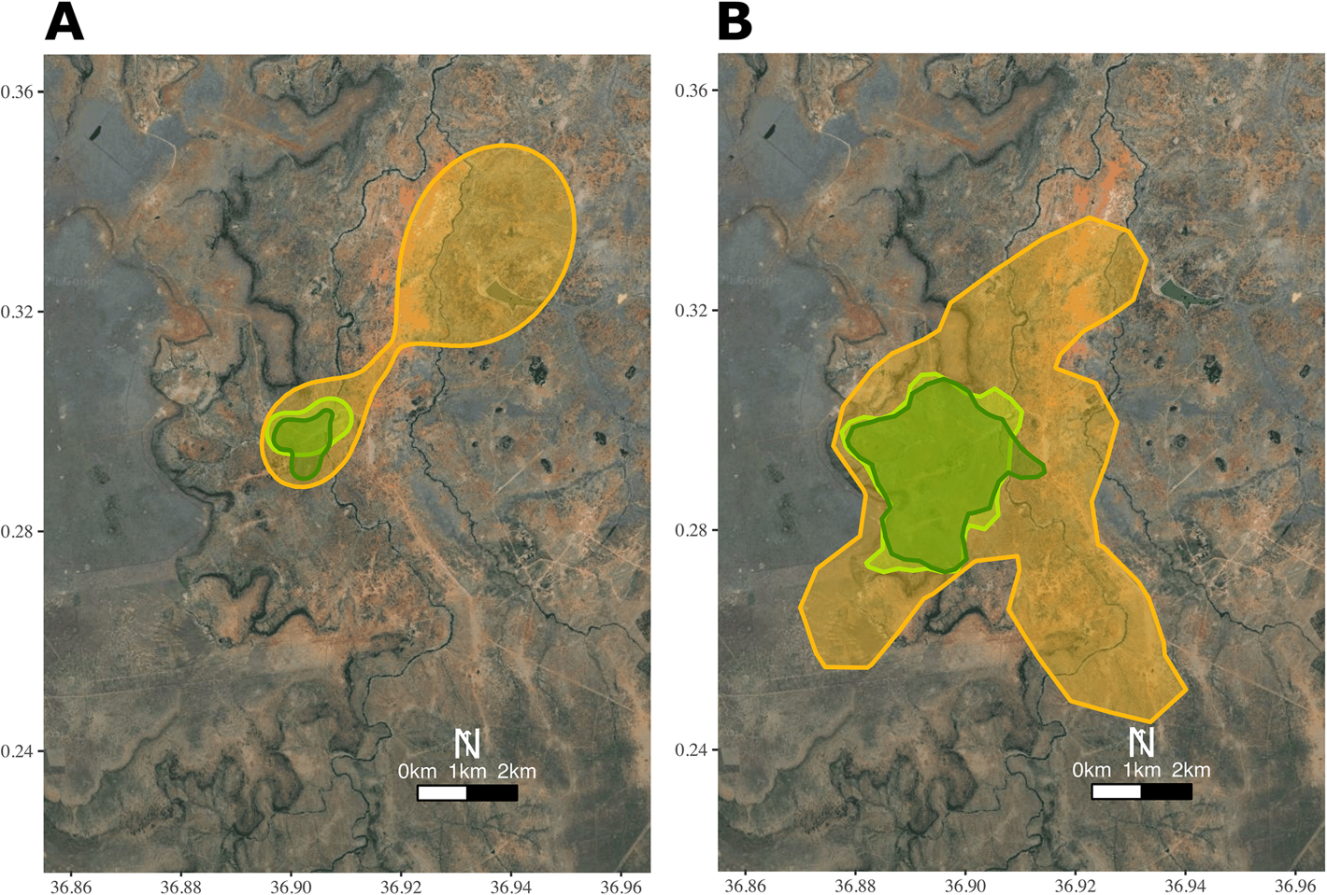
Example of shifts in home-range in response to seasonality. **A** During drought (yellow, Season 6) one of our study groups used a much larger area than in a preceding intermediate season (light green, Season 4) or than in the wet season that followed the drought (dark green, Season 7). Polygons represent the 95% AKDE. **B** Seasons and colours as in (**A**) but polygons represent space use by the entire population. Polygons represent the 95% KDE. Base imagery was downloaded from GoogleMaps (Map data ©2021 Google)

We found that seasonal variation in environmental conditions corresponded to significant differences in collective movement patterns. In dry conditions, groups of vulturine guineafowl used much larger areas and travelled longer distances on a daily basis, potentially in the search of resources. We also found evidence that groups had lower day-to-day fidelity in their movement during breeding seasons than during dry seasons. However, groups had greater overlap in dry-to-dry and wet-to-wet seasonal home ranges, and less overlap between seasons comprising different conditions (e.g. dry and wet, suggesting that groups had to shift their home ranges in response to changing conditions (Fig. 3). Our visual observations confirm that when drought conditions become extreme, groups start to travel long distances to larger water bodies, such as the local river, where they are also more likely to encounter other groups [32]. Overall, these results highlight how seasonality drives changes in the expression of collective actions of moving animal groups. Finally, we found that these changes correspond to major changes at the level of the population, with the overall area used by all groups combined more than tripling during the dry seasons.

In addition to environmental factors, group size [49] and group composition [50, 51] are known to impact group behaviours. During intermediate seasons, guineafowl groups of intermediate size exhibit larger home range size by travelling shorter daily distances and by exploring new areas day by day, than larger or smaller groups [26]. However, our present study suggests that the effects of seasonality might be more pronounced than those of group size—the difference between seasons was often substantially greater than the variation in group movement properties within seasons (with the exception of wet-breeding seasons). Further, group size might lay a different role on ranging behaviour in different seasons. For example, larger groups may spread across larger areas during wet-breeding season, as more females move to different areas to nest, which is a different mechanism to what generates differences in area used by groups during dry seasons [26]. Future work looking specifically at drivers of variation in movement across groups within seasons might consider the contributions of different factors, including climatic conditions and resource distribution.

Our study benefited from using identical methods to track the movements of replicated groups and from tracking the same groups across seasons. This was important to be able to model how seasons affected each group because we could collect identical data across groups and across each study period. Focusing on individual movements—as opposed to whole group tracking—as a proxy for group movement, even in a species such as the vulturine guineafowl that groups move very cohesively, might underestimate home-range size if not all group members reach the boundaries in a group’s range. However, we found that groups re-used the same areas consistently, and previous work [32] has shown that group members remain in close proximity almost all of the time. Further, we conducted an a posteriori test by adding the number of individuals tracked in each group, and found that this was not a significant predictor of home range size (Supplementary Table 7).

Coordinated collective behaviour can emerge from local interactions between individuals in the absence of centralised control [52]. Even in natural groups consisting of members with different social status (e.g. dominance rank, state of need, experience) and varying characteristics (e.g. sex, body size, age), individuals seem to follow simple rules to coordinate collective actions [53, 54]. For example, to remain cohesive meerkats follow the most vocally active spot of their group [55] and wild olive baboons coordinate with their nearest neighbours [53]. What remains largely unexplored is whether these individual rules can allow groups to adjust their behaviour to changing environments. The seasonal differences we found in ranging behaviour and cohesion might indicate either that the interaction rules among individuals change in response to seasonality to allow groups to adapt to new conditions [9], or that the existing rules can allow groups to achieve substantially different outcomes across different environmental conditions through the emergent properties of inter-individual interactions.

Collective decision-making mechanisms can potentially allow groups to adjust to changing environments without requiring individuals to change their decision rules. For example, who contributes to group decisions can be dictated by differences among individuals in their physiology or state of need, which could mean that hungry or thirsty individuals can emerge as leaders of their group and bring the group to forage more [56, 57]. Similarly, individual information state could shift under different conditions, with some individuals becoming more influential than others [58]. For example, older female elephants can lead their group to rarely-visited resources during periods of extreme droughts [7]. Thus, even with shared decision-making (i.e. all members have equal ability to influence where the group goes [59, 60];, one individual can decide for the whole group if it has important information (i.e. the older and more knowledgeable elephants show the way [61, 62];). The propensity for an individual to lead the group might be a function of both the state of the individual and the environmental conditions, with shared decision-making providing a flexible algorithm for collective action. However, whether the ability for knowledgeable individuals to become leaders is also linked to their social status remains largely unexplored. For example, can an individual that has recently migrated from its natal group lead their new group to a resource that only it knows about? The links between environmental conditions, collective movement and decision-making represent a rich area for future research.

A further question is how individuals themselves manage their energy budgets across different seasons, and whether being in a group facilitates or hinders this. We have shown that during extreme droughts, groups of vulturine guineafowl, which remain stable [32], travel farther. This pattern is predicted by groups having a greater ability to deplete resources [21] and requiring more overall resources to satisfy all group members. However, travelling itself requires energy [63] and imposes higher risk of hyperthermia and dehydration, especially during drought. Recent work on vulturine guineafowl demonstrated that dispersing subadults moved substantially more efficiently during transience, thereby achieving much larger daily displacements with little effects on their energy budget [64]. Changes in individual physiology (e.g. facultative hyperthermia [65]; metabolic water production through fat [66]) may also allow group members to perform long daily movements while buffering the negative effects of environmental conditions. It remains to be determined whether group dynamics are impacted by needs of homeostasis maintenance and if the costs of the meteorological environment can be buffered.

It also remains to be examined whether there is a feedback from group-level processes onto individual-level physiological state. When groups make a decision, not all individuals benefit equally [34, 67], with some paying a ‘consensus cost’ to remaining with the group. Consensus decisions may be suboptimal for an individual given its current needs (for example to move on from a foraging patch before the individual has eaten enough [34]). A major question is whether there is seasonal variation in the consensus costs that group members pay. That is, do some individuals miss out on food more during dry seasons, and are there therefore greater costs in drier than in wetter seasons? Such changes are presumed to also underlie seasonal changes in the social system in many species [68]. The interplay between individual physiology, seasonal variation in resource distribution, and collective decision-making that allow individuals to maintain group cohesion while covering their daily needs is an exciting focus for future studies.

## Conclusions

Severe and extended droughts, such as the East-African drought of early 2019 [69], are predicted to occur more frequently due to climate change [23]. During this particular drought (seasons 5 and 6 in our data), we observed that vulturine guineafowl groups significantly changed their ranging patterns in our study area. They ranged over larger areas and, correspondingly, travelled longer distances every day. Further, many groups used different areas to their normal ranges (Fig. 3). Such behavioural strategies are expected to be critical to buffer the effects of climate changes on individual fitness [70] and, in turn, influence the population responses to these changes [71]. However, by forcing group-living species to travel longer distances and into new areas, droughts might impact individual survival, in turn leading to population-level shrinkage [72]. We found that the outcome of group changes in behaviour resulted in a major increase in the area needed for the whole population. Our findings potentially capture an important process for savannah-dwelling species: as animal groups range into wider areas to cover their needs, they could start overlapping more with human populations, which can lead to a range of human-wildlife conflicts [73]. Thus, species may be more limited by dry-season space availability than they are by resources needed to breed.

It is critical to understand how groups can achieve changes in their behaviour across the increasingly large spectrum of environmental conditions they experience. Specifically, does the same set of interaction rules provide sufficient flexibility for groups to adapt to such large differences in environmental conditions or do they adopt different rules in order to attain such different goals? Studying these processes will allow us to better understand not only how collective outcomes are driven by the environment, but whether these outcomes are the result of changes in the way individuals interact with each other. In doing so, we can also gain a better understanding of how population-level responses to climate change can emerge from the interactions among individuals, thereby linking processes taking place at the individual-level with those that play out across ecological landscapes. Such knowledge is imperative for making robust predictions about how global environmental change will impact animal societies.

## Supplementary Information

**Additional file 1: Supplementary Table 1.** Details of the eleven two-month seasons that formed part of the study. Cumulative rainfall per season was collected from a weather station at the Mpala Research Centre [4]. Average seasonal NDVI was calculated as explained in the Supplementary Text. Seasons were divided in three categories based both on NDVI on glades and the total rainfall: wet, intermediate and dry. Dry corresponds to a severe drought that occurred across Kenya in 2019. Deaths, tag losses and new trapping sessions result in differences in the number of individuals (range 22 to 40) that were tracked in each season. **Supplementary Table 2.** Results of the LMM for the core home range size 50% and its response to seasonality. Reference level of season type is set to dry. **Supplementary Table 3.** Results of the LMM for home range size 95% and its response to seasonality. Reference level of season type is set to dry. **Supplementary Table 4.** Results of the LMM for daily travel distance and its response to seasonality. Reference level of season type is set to dry. **Supplementary Table 5.** Results of the LMM for day-to-day site fidelity and its response to seasonality. Reference level of season type is set to dry seasons. **Supplementary Table 6.** Results of the LMM for seasonal range overlap and its response to seasonality. Reference level of season type is set to the overlap between two dry seasons. **Supplementary Table 7.** Results of the LMM, in which we added the number of individuals tracked in each group in each season as a predictor for home range, alongside with the fixed and random effects of the LMM presented in Supplementary Table 2. We found that the number of individuals tracked in each group was not a significant predictor of home range size. Reference level of season type is set to dry. **Supplementary Figure 1.** Average NDVI on the glades, which are typical foraging areas for vulturine guineafowl, and total rainfall per day for each of the study periods. The dashed grey lines represent a period from which rainfall data wass missing. **Supplementary Figure 2.** The 50 and 95% AKDEs, their confidence intervals and the distribution of GPS detections for one group in three seasons; **(A)** intermediate season (light green polygon in Fig. 3, Season 4), **(B)** drought (yellow in Fig. 3, Season 6) and **(C)** wet season that followed the drought (dark green, Season 7). Circles represent errors in the data.

## Data Availability

All data used in this study are stored on Movebank under the study name Avulturinum_Farine: https://www.movebank.org/cms/webapp?gwt_fragment=page=studies,path=study475851705. The code to run the analysis and to replicate the results of the models, together with the input data of the models, is available on: https://github.com/DanPapageorgiou/ranging_seasonality

## References

[1] Lindström Å, Chapman BB, Jonzén N, Klaassen M. Movement and migration in a changing world. Animal Movement Across Scales. Oxford University Press; 2014:36–50. Available from: 10.1093/acprof:oso/9780199677184.001.0001

[2] Rozen-Rechels D, Dupoué A, Lourdais O, Chamaillé-Jammes S, Meylan S, Clobert J, le Galliard JF (2019). When water interacts with temperature: ecological and evolutionary implications of thermo-hydroregulation in terrestrial ectotherms. Ecol Evol.

[3] Mitchell D, Snelling EP, Hetem RS, Maloney SK, Strauss WM, Fuller A (2018). Revisiting concepts of thermal physiology: Predicting responses of mammals to climate change. Wang D, editor. J Anim Ecol.

[4] Prox L, Farine DR (2020). A framework for conceptualizing dimensions of social organization in mammals. Ecol Evol.

[5] Long RA, Bowyer RT, Porter WP, Mathewson P, Monteith KL, Kie JG (2014). Behavior and nutritional condition buffer a large-bodied endotherm against direct and indirect effects of climate. Ecol Monogr.

[6] Cunningham SJ, Martin RO, Hockey PAR (2015). Can behaviour buffer the impacts of climate change on an arid-zone bird?. Ostrich..

[7] Foley C, Pettorelli N, Foley L (2008). Severe drought and calf survival in elephants. Biol Lett.

[8] Prabhakar B, Dektar KN, Gordon DM (2012). The regulation of ant Colony foraging activity without spatial information. PLoS Comput Biol.

[9] Gordon DM (2016). The evolution of the algorithms for collective behavior. Cell Syst.

[10] Couzin ID, Ioannou CC, Demirel G, Gross T, Torney CJ, Hartnett A, et al. Uninformed individuals promote democratic consensus in animal groups. Science. 2011;334:1578–80. https://science.sciencemag.org/content/334/6062/1578. 10.1126/science.1210280 22174256

[11] Strandburg-Peshkin A, Farine DR, Couzin ID, Crofoot MC (2015). Shared decision-making drives collective movement in wild baboons. Supplementary material. Science.

[12] Krause J, Ruxton G. Living in groups: Oxford University Press; 2002.

[13] Couzin ID (2006). Behavioral ecology: social Organization in Fission–Fusion Societies. Curr Biol.

[14] Aureli F, Schaffner CM, Boesch C, Bearder SK, Call J, Chapman CA, Connor R, Fiore AD, Dunbar RIM, Henzi SP, Holekamp K, Korstjens AH, Layton R, Lee P, Lehmann J, Manson JH, Ramos-Fernandez G, Strier KB, Schaik CP (2008). Fission-fusion dynamics new research frameworks. Curr Anthropol.

[15] Clutton-Brock T, Manser M, Koenig W, Dickinson J (2016). Meerkats: cooperative breeding in the Kalahari. Coop breed Vertebr stud Ecol Evol Behav.

[16] Dunbar RIM (1992). Time: a hidden constraint on the behavioural ecology of baboons. Behav Ecol Sociobiol.

[17] Paniw M, Maag N, Cozzi G, Clutton-Brock T, Ozgul A (2019). Life history responses of meerkats to seasonal changes in extreme environments. Science.

[18] Thackeray SJ, Henrys PA, Hemming D, Bell JR, Botham MS, Burthe S (2016). Phenological sensitivity to climate across taxa and trophic levels. Nature.

[19] Rubenstein DR (2007). Territory quality drives intraspecific patterns of extrapair paternity. Behav Ecol.

[20] Harestad AS, Bunnel FL (1979). Home range and body weight--a reevaluation. Ecology.

[21] Giraldeau L-A, Caraco T. Social foraging theory: Princeton University Press; 2018.

[22] Owen-Smith N, Fryxell JM, Merrill EH (2010). Foraging theory upscaled: the behavioural ecology of herbivore movement. Philos Trans R Soc B Biol Sci.

[23] Trenberth KE, Dai A, van der Schrier G, Jones PD, Barichivich J, Briffa KR, et al. Global warming and changes in drought. Nat Clim Chang. 2014;4(1):17–22. https://www.nature.com/articles/nclimate2067.

[24] Berdahl A, Torney CJ, Ioannou CC, Faria JJ, Couzin ID (2013). emergent sensing of complex environments by mobile animal groups. Science (80- ).

[25] Biro D, Sasaki T, Portugal SJ (2016). Bringing a time–depth perspective to collective animal behaviour. Trends Ecol Evol.

[26] Papageorgiou D, Farine DR. Group size and composition influence collective movement in a highly social terrestrial bird. eLife. 2020;9:e59902. https://elifesciences.org/articles/59902. 10.7554/eLife.59902 PMC7655099 33168135

[27] Börger L, Dalziel BD, Fryxell JM (2008). Are there general mechanisms of animal home range behaviour? A review and prospects for future research. Ecol Lett.

[28] Johnson C, Piel AK, Forman D, Stewart FA, King AJ (2015). The ecological determinants of baboon troop movements at local and continental scales. Mov Ecol.

[29] Boyer D, Crofoot MC, Walsh PD (2012). Non-random walks in monkeys and humans. J R Soc Interface.

[30] Noonan MJ, Tucker MA, Fleming CH, Akre TS, Alberts SC, Ali AH (2019). A comprehensive analysis of autocorrelation and bias in home range estimation. Ecol Monogr.

[31] King AJ, Fehlmann G, Biro D, Ward AJ, Fürtbauer I. Re-wilding Collective Behaviour: An Ecological Perspective. Trends Ecol Evol. Elsevier Ltd; 2018;33:347–57. Available from: http://www.cell.com/trends/ecology-evolution/fulltext/S0169-5347(18)30055-7. 10.1016/j.tree.2018.03.004 29627203

[32] Papageorgiou D, Christensen C, Gall GEC, Klarevas-Irby JA, Nyaguthii B, Couzin ID, Farine DR (2019). The multilevel society of a small-brained bird. Curr Biol.

[33] Guindre-Parker S, Rubenstein DR (2020). Survival benefits of group living in a fluctuating environment. Am Nat.

[34] Papageorgiou D, Farine DR (2020). Shared decision-making allows subordinates to lead when dominants monopolize resources. Sci Adv.

[35] Naude VN, Smyth LK, Weideman EA, Krochuk BA, Amar A (2019). Using web-sourced photography to explore the diet of a declining African raptor, the Martial Eagle (*Polemaetus bellicosus*). Condor.

[36] Pettorelli N, Vik JO, Mysterud A, Gaillard J-M, Tucker CJ, Stenseth NC (2005). Using the satellite-derived NDVI to assess ecological responses to environmental change. Trends Ecol Evol.

[37] Caylor KK, Gitonga J, Martins DJ (2020). Mpala research Centre meteorological and hydrological dataset.

[38] Csardi G, Nepusz T (2006). The igraph software package for complex network research. InterJournal.

[39] Clauset A, Newman MEJ, Moore C (2004). Finding community structure in very large networks. Phys Rev E.

[40] Liechti JI, Bonhoeffer S (2019). A time resolved clustering method revealing longterm structures and their short-term internal dynamics. arXiv.

[41] Fleming CH, Fagan WF, Mueller T, Olson KA, Leimgruber P, Calabrese JM (2015). Rigorous home range estimation with movement data: a new autocorrelated kernel density estimator. Ecology.

[42] Calabrese JM, Fleming CH, Gurarie E (2016). Ctmm: an R package for analyzing animal relocation data as a continuous-time stochastic process. Methods Ecol Evol.

[43] Fleming CH, Calabrese JM (2018). ctmm: Continuous-Time Movement Modeling. R Packag. version 0.5.1.

[44] Winner K, Noonan MJ, Fleming CH, Olson KA, Mueller T, Sheldon D (2018). Statistical inference for home range overlap. Scales K, editor. Methods Ecol Evol.

[45] Calenge C (2006). The package “adehabitat” for the R software: a tool for the analysis of space and habitat use by animals. Ecol Model.

[46] Bates D, Mächler M, Bolker B, Walker S (2015). Fitting linear mixed-effects models using lme4. J Stat Softw.

[47] R Core Team (2018). R: a language and environment for statistical computing.

[48] Kuznetsova A, Brockhoff PB, Christensen RHB (2017). lmerTest package: tests in linear mixed effects models. J Stat Softw.

[49] Cantor M, Aplin LM, Farine DR. A primer on the relationship between group size and group performance. Anim Behav. 2020;166:139–46. Available from: https://linkinghub.elsevier.com/retrieve/pii/S0003347220301767.

[50] Farine DR, Montiglio PO, Spiegel O (2015). From individuals to groups and Back: the evolutionary implications of group phenotypic composition. Trends Ecol Evol.

[51] Aplin LM, Farine DR, Mann RP, Sheldon BC (2014). Individual-level personality influences social foraging and collective behaviour in wild birds. Proc R Soc B.

[52] Couzin ID, Krause J, James R, Ruxton GD, Franks NR (2002). Collective memory and spatial sorting in animal groups. J Theor Biol.

[53] Farine DR, Strandburg-Peshkin A, Berger-Wolf T, Ziebart B, Brugere I, Li J (2016). Both nearest neighbours and long-term affiliates predict individual locations during collective movement in wild baboons. Sci Rep.

[54] Farine DR, Strandburg-Peshkin A, Couzin ID, Berger-Wolf TY, Crofoot MC (2017). Individual variation in local interaction rules can explain emergent patterns of spatial organization in wild baboons. Proc R Soc B Biol Sci.

[55] Gall GEC, Manser MB (2017). Group cohesion in foraging meerkats: follow the moving ‘vocal hot spot’. R Soc Open Sci.

[56] Smith JE, Estrada JR, Richards HR, Dawes SE, Mitsos K, Holekamp KE (2015). Collective movements, leadership and consensus costs at reunions in spotted hyaenas. Anim Behav.

[57] Conradt L, Krause J, Couzin ID, Roper TJ (2009). “Leading according to need” in self-organizing groups. Am Nat.

[58] Reebs S (2000). Can a minority of informed leaders determine the foraging movements of a fish shoal?. Anim Behav.

[59] Conradt L, Roper T (2007). Democracy in animals: the evolution of shared group decisions. Proc R Soc B Biol Sci.

[60] Strandburg-Peshkin A, Papageorgiou D, Crofoot MC, Farine DR (2018). Inferring influence and leadership in moving animal groups. Philos Trans R Soc B Biol Sci.

[61] Brent LJN, Franks DW, Foster EA, Balcomb KC, Cant MA, Croft DP. Ecological Knowledge, Leadership, and the Evolution of Menopause in Killer Whales. Curr Biol. 2015;25:746–50. Available from: 10.1016/j.cub.2015.01.037. 10.1016/j.cub.2015.01.037 25754636

[62] Allen CRB, Brent LJN, Motsentwa T, Weiss MN, Croft DP (2020). Importance of old bulls: leaders and followers in collective movements of all-male groups in African savannah elephants (Loxodonta africana). Sci Rep.

[63] Halsey LG (2016). Terrestrial movement energetics: current knowledge and its application to the optimising animal. J Exp Biol.

[64] Klarevas-Irby JA, Wikelski M, Farine DR. Efficient movement strategies mitigate the energetic cost of dispersal. Ecol Lett. 2021;24(7):1432–42. 10.1111/ele.13763. 10.1111/ele.13763 33977638

[65] Gerson AR, McKechnie AE, Smit B, Whitfield MC, Smith EK, Talbot WA (2019). The functional significance of facultative hyperthermia varies with body size and phylogeny in birds. Funct Ecol.

[66] MacMillen RE (1990). Water economy of granivorous birds: a predictive model. Condor.

[67] King AJ, Douglas CMS, Huchard E, Isaac NJB, Cowlishaw G (2008). Dominance and affiliation mediate despotism in a social primate. Curr Biol.

[68] Schreier AL, Swedell L (2012). Ecology and sociality in a multilevel society: ecological determinants of spatial cohesion in hamadryas baboons. Am J Phys Anthropol.

[69] National Drought Management Authority of Kenya (2019). National drought early warning bulletin for May 2019.

[70] Huey RB, Kearney MR, Krockenberger A, Holtum JAM, Jess M, Williams SE (2012). Predicting organismal vulnerability to climate warming: roles of behaviour, physiology and adaptation. Philos Trans R Soc B Biol Sci.

[71] Wong BBM, Candolin U (2015). Behavioral responses to changing environments. Behav Ecol.

[72] Bourne AR, Cunningham SJ, Spottiswoode CN, Ridley AR. Hot droughts compromise interannual survival across all group sizes in a cooperatively breeding bird. Pinter-Wollman N, editor. Ecol Lett. 2020;23(12):1776–88. Available from: 10.1111/ele.13604. 10.1111/ele.13604 32945068

[73] Mukeka JM, Ogutu JO, Kanga E, Røskaft E (2019). Human-wildlife conflicts and their correlates in Narok County, Kenya. Glob Ecol Conserv.

