# Supplementary material for: Seasonality impacts collective movements in a wild group-living bird
Source: Mov Ecol. 2021 Jul 8;9:38. doi: 10.1186/s40462-021-00271-9 (PMC8268463; doi:10.1186/s40462-021-00271-9)
Supplement: Supplementary file 1 — Additional file 1: Supplementary Table 1. Details of the eleven two-month seasons that formed part of the study. Cumulative rainfall per season was collected from a weather station at the Mpala Research Centre [4]. Average seasonal NDVI was calculated as explained in the Supplementary Text. Seasons were divided in three categories based both on NDVI on glades and the total rainfall: wet, intermediate and dry. Dry corresponds to a severe drought that occurred across Kenya in 2019. Deaths, tag losses and new trapping sessions result in differences in the number of individuals (range 22 to 40) that were tracked in each season. Supplementary Table 2. Results of the LMM for the core home range size 50% and its response to seasonality. Reference level of season type is set to dry. Supplementary Table 3. Results of the LMM for home range size 95% and its response to seasonality. Reference level of season type is set to dry. Supplementary Table 4. Results of the LMM for daily travel distance and its response to seasonality. Reference level of season type is set to dry. Supplementary Table 5. Results of the LMM for day-to-day site fidelity and its response to seasonality. Reference level of season type is set to dry seasons. Supplementary Table 6. Results of the LMM for seasonal range overlap and its response to seasonality. Reference level of season type is set to the overlap between two dry seasons. Supplementary Table 7. Results of the LMM, in which we added the number of individuals tracked in each group in each season as a predictor for home range, alongside with the fixed and random effects of the LMM presented in Supplementary Table 2. We found that the number of individuals tracked in each group was not a significant predictor of home range size. Reference level of season type is set to dry. Supplementary Figure 1. Average NDVI on the glades, which are typical foraging areas for vulturine guineafowl, and total rainfall per day for each of the study periods. The dashe [file 40462_2021_271_MOESM1_ESM.docx]

Supplementary material for :

Seasonality impacts collective movement in a wild group-living bird

Danai Papageorgiou^1,2,3,4,5^, David Rozen-Rechels^1,2,3,4^, Brendah Nyaguthii^6,7,8^, Damien Farine^1,3,4,8^

1. Max Planck Institute of Animal Behavior, Department of Collective Behavior, Universitätsstraße 10, Konstanz, 78457, Germany

2. University of Konstanz, Department of Biology, Universitätsstraße 10, Konstanz, 78457, Germany

3. University of Konstanz, Center for the Advanced Study of Collective Behaviour, Universitätsstraße 10, Konstanz, 78457, Germany

4. University of Zurich, Department of Evolutionary Biology and Environmental Studies, Winterthurerstrasse 190, 8057 Zurich, Switzerland

5. Kenya Wildlife Service, P.O. Box 40241-001000, Nairobi, Kenya

6. University of Eldoret, School of Natural Resource Management, Department of

Wildlife, 1125-30100 Eldoret, Kenya

7. Mpala Research Center, P.O. Box 92, Nanyuki, 10400, Kenya.

8. Department of Ornithology, National Museums of Kenya, P.O. Box 40658-001000, Nairobi, Kenya

**Supplementary Text**

*Calculation of Normalized Difference of Vegetation Index (NDVI)*

In order to calculate average NDVI in the landscape, we downloaded all satellite images (L1C products) from the Sentinel-2 mission [1] over the study period (tile 37N BA). The Sentinel-2 mission is composed of two satellites equipped with multi-spectral optical sensors, capturing a raster image of each tile with a frequency of 10 days. We then obtained an image of the zone of interest every 5 days (142 rasters). We corrected for distortion of atmosphere, terrain or cirrus clouds using the Sen2Cor v2.8 tool in command line (<https://step.esa.int/main/snap-supported-plugins/sen2cor/>) from the SNAP Toolbox version 8.00 [2] with default parameters. We then resampled the product at a resolution of 10m and subset the raster image to the extent of the zone studied. We focused on bands measuring Near-InfraRed (NIR, band 8A) and Red (band 4) bands as well as the classification band for cloud masking purposes. We imported the stack of rasters into R with the ‘raster’ package and cropped them to the shape of the maximum observed range of the population (Fig. 3B). We then masked all pixels corresponding to clouds (cloud medium probability, cloud high probability, thin cirrus, cloud shadows) or other elements, often linked to clouds, that could bias the analysis (snow or ice, dark features, unclassified), based on the classification band. Rasters in which the cover of such features exceeded 20% were removed from the analyses to avoid bias due to missing data (71 rasters remaining). We then calculated NDVI for each pixel following the equation[3]:

$$\text{NDVI=}\frac{\text{NIR}-\text{Red}}{\text{NIR+Red}}$$

NDVI values range from -1 to 1. Negative values are generally for surfaces other than soil and vegetation (water). Naked soil values are around 0. Positive values, starting around 0.1, are for vegetation cover. The higher the NDVI, the denser and the greener is the cover (high NIR reflectance compared to Red). We calculated the average NDVI for pixels included in the glades (typical foraging areas for our study species) for each date available (Supplementary Figure 1).

**Supplementary Table 1.** Details of the eleven two-month seasons that formed part of the study. Cumulative rainfall per season was collected from a weather station at the Mpala Research Centre [4]. Average seasonal NDVI was calculated as explained in the Supplementary Text. Seasons were divided in three categories based both on NDVI on glades and the total rainfall: wet, intermediate and dry. Dry corresponds to a severe drought that occurred across Kenya in 2019. Deaths, tag losses and new trapping sessions result in differences in the number of individuals (range 22 to 40) that were tracked in each season.

|  |  |  |  |  |  |  |  | **Population ranging** | |
| --- | --- | --- | --- | --- | --- | --- | --- | --- | --- |
| **Season** | **Start Date** | **End Date** | **Total Rain  (mm)** | **Average**  **seasonal**  **NDVI** | **Season  type** | **Groups** | **Individuals** | **50% KDE** | **95% KDE** |
| 1 | 01/05/2018 | 29/06/2018 | 180 | 0.572 | wet-breeding | 14 | 22 | 1.994 | 7.003 |
| 2 | 30/06/2018 | 28/08/2018 | 31 | 0.419 | wet | 14 | 23 | 2.159 | 7.151 |
| 3 | 29/08/2018 | 27/10/2018 | NA | 0.294 | intemediate | 12 | 25 | 1.794 | 8.238 |
| 4 | 28/10/2018 | 26/12/2018 | 22 | 0.389 | intemediate | 15 | 37 | 1.929 | 8.856 |
| 5 | 27/12/2018 | 24/02/2019 | 8 | 0.273 | dry | 14 | 35 | 6.707 | 25.988 |
| 6 | 25/02/2019 | 26/04/2019 | 14 | 0.206 | dry | 16 | 33 | 7.648 | 37.546 |
| 7 | 27/04/2019 | 25/06/2019 | 162 | 0.420 | wet | 16 | 41 | 1.777 | 7.837 |
| 8 | 26/06/2019 | 24/08/2019 | 100 | 0.483 | wet | 15 | 40 | 1.812 | 6.515 |
| 9 | 25/08/2019 | 23/10/2019 | 148 | 0.389 | wet | 18 | 34 | 1.753 | 7.188 |
| 10 | 24/10/2019 | 22/12/2019 | 210 | 0.539 | wet-breeding | 26 | 38 | 1.797 | 7.788 |
| 11 | 23/12/2019 | 20/02/2020 | 67 | 0.475 | wet | 22 | 36 | 1.893 | 9.229 |

**Supplementary Table 2.** Results of the LMM for the core home range size 50% and its response to seasonality. Reference level of season type is set to dry.

| **Home Range Size 50%** | | | | | |
| --- | --- | --- | --- | --- | --- |
|  | **Estimate** | **Std. Error** | **df** | **t value** | **Pr(>\|t\|)** |
| **Intercept** | 9.785 | 0.726 | 163.909 | 13.471 | **<0.001** |
| **Season type: Intermediate** | -8.344 | 0.944 | 313.676 | -8.836 | **<0.001** |
| **Season type: Wet** | -8.586 | 0.803 | 320.218 | -10.698 | **<0.001** |
| **Season type: Wet-breeding** | -8.590 | 0.936 | 319.992 | -9.174 | **<0.001** |
| **Scaled days tracked** | 0.610 | 0.301 | 314.299 | 2.025 | **0.044** |
| ***Random effects*** | | | |  |  |
| **Groups** | **Name** | **Variance** | **Std.dev** |  |  |
| **Group ID** | **Intercept** | 1.304 | 1.142 |  |  |
| **Residual** |  | 21.490 | 4.636 |  |  |

**Supplementary Table 3.** Results of the LMM for home range size 95% and its response to seasonality. Reference level of season type is set to dry.

| **Home Range Size 95%** | | | | | |
| --- | --- | --- | --- | --- | --- |
|  | **Estimate** | **Std. Error** | **df** | **t value** | **Pr(>\|t\|)** |
| **Intercept** | 17.229 | 1.260 | 155.880 | 13.677 | **<0.001** |
| **Season type: Intermediate** | -14.480 | 1.632 | 312.771 | -8.872 | **<0.001** |
| **Season type: Wet** | -14.637 | 1.387 | 319.885 | -10.551 | **<0.001** |
| **Season type: Wet-breeding** | -14.852 | 1.618 | 319.659 | -9.177 | **<0.001** |
| **Scaled days tracked** | 0.199 | 0.521 | 313.447 | 0.382 | 0.702 |
| ***Random effects*** | | | |  |  |
| **Groups** | **Name** | **Variance** | **Std.dev** |  |  |
| **Group ID** | **Intercept** | 4.083 | 2.021 |  |  |
| **Residual** |  | 64.163 | 8.010 |  |  |

**Supplementary Table 4.** Results of the LMM for daily travel distance and its response to seasonality. Reference level of season type is set to dry.

| **Daily Travel Distance** | | | | | | | |
| --- | --- | --- | --- | --- | --- | --- | --- |
|  | **Estimate** | **Std. Error** | **df** | **t value** | | **Pr(>\|t\|)** |  |
| **Intercept** | 11.875 | 0.246 | 114.154 | 48.215 | | **<0.001** |  |
| **Season type: Intermediate** | -2.752 | 0.311 | 306.479 | -8.837 | | **<0.001** |  |
| **Season type: Wet** | -2.694 | 0.265 | 317.279 | -10.161 | | **<0.001** |  |
| **Season type:**  **Wet-breeding** | -3.083 | 0.309 | 317.169 | -9.968 | | **<0.001** |  |
| **Scaled days tracked** | 0.455 | 0.099 | 307.496 | 4.578 | | **<0.001** |  |
| ***Random effects*** | | | | |  |  |  |
| **Groups** | **Name** | **Variance** | **Std.dev** |  | |  |  |
| **Group ID** | **Intercept** | 0.200 | 0.447 |  | |  |  |
| **Residual** |  | 2.332 | 1.527 |  | |  |  |

**Supplementary Table 5.** Results of the LMM for day-to-day site fidelity and its response to seasonality. Reference level of season type is set to dry seasons.

| **Day-to-day site fidelity** | | | | | |
| --- | --- | --- | --- | --- | --- |
|  | **Estimate** | **Std. Error** | **df** | **t value** | **Pr(>\|t\|)** |
| **Intercept** | 0.905 | 0.014 | 103.425 | 65.810 | **<0.001** |
| **Season type: Intermediate** | -0.018 | 0.017 | 117.137 | -1.073 | 0.285 |
| **Season type: Wet** | -0.022 | 0.014 | 119.433 | -1.578 | 0.117 |
| **Season type: Wet-breeding** | -0.054 | 0.016 | 123.238 | -3.401 | **0.001** |
| **Days tracked** | 0.014 | 0.005 | 118.813 | 2.907 | **0.004** |
| ***Random effects*** | | | |  |  |
| **Groups** | **Name** | **Variance** | **Std.dev** |  |  |
| **ID** | **Intercept** | 0.001 | 0.030 |  |  |
| **Residual** |  | 0.003 | 0.051 |  |  |

**Supplementary Table 6.** Results of the LMM for seasonal range overlap and its response to seasonality. Reference level of season type is set to the overlap between two dry seasons.

| **Seasonal Range Fidelity** | | | | | |
| --- | --- | --- | --- | --- | --- |
|  | **Estimate** | **Std. Error** | **df** | **t value** | **Pr(>\|t\|)** |
| **Intercept** | 0.777 | 0.070 | 231.607 | 11.090 | **<0.001** |
| **Season types: dry to intermediate** | -0.277 | 0.075 | 230.636 | -3.718 | **<0.001** |
| **Season types: dry to wet** | -0.293 | 0.087 | 232.892 | -3.350 | **0.001** |
| **Season types: dry to wet-breeding** | -0.332 | 0.086 | 232.336 | -3.851 | **<0.001** |
| **Season types: intermediate to intermediate** | 0.125 | 0.096 | 233.234 | 1.300 | 0.195 |
| **Season types: intermediate to wet** | 0.152 | 0.086 | 235.442 | 1.768 | 0.078 |
| **Season types: intermediate to wet-breeding** | 0.108 | 0.085 | 234.632 | 1.265 | 0.207 |
| **Season types: wet to wet** | 0.004 | 0.072 | 235.469 | 0.062 | 0.951 |
| **Season types: wet to wet-breeding** | 0.067 | 0.072 | 236.092 | 0.924 | 0.356 |
| ***Random effects*** | | | |  |  |
| **Groups** | **Name** | **Variance** | **Std.dev** |  |  |
| **ID** | **Intercept** | 0.008 | 0.091 |  |  |
| **Residual** |  | 0.048 | 0.219 |  |  |

**Supplementary Table 7.** Results of the LMM, in which we added the number of individuals tracked in each group in each season as a predictor for home range, alongside with the fixed and random effects of the LMM presented in Supplementary Table 2. We found that the number of individuals tracked in each group was not a significant predictor of home range size. Reference level of season type is set to dry.

|  | **Estimate** | **Std. Error** | **df** | **t value** | **Pr(>\|t\|)** |
| --- | --- | --- | --- | --- | --- |
| **Intercept** | 10.255 | 0.943 | 213.644 | 10.876 | **<0.001** |
| **Season type: Intermediate** | -8.344 | 0.944 | 312.774 | -8.836 | **<0.001** |
| **Season type: Wet** | -8.624 | 0.804 | 319.370 | -10.726 | **<0.001** |
| **Season type: Wet-breeding** | -8.705 | 0.947 | 318.992 | -9.192 | **<0.001** |
| **Scaled days tracked** | 0.603 | 0.302 | 314.010 | 2.000 | **0.046** |
| **Number of individuals  per group per season** | -0.183 | 0.233 | 112.831 | -0.786 | 0.434 |
| ***Random effects*** | | | |  |  |
| **Groups** | **Name** | **Variance** | **Std.dev** |  |  |
| **Group ID** | **Intercept** | 1.385 | 1.177 |  |  |
| **Residual** |  | 21.480 | 4.635 |  |  |

**
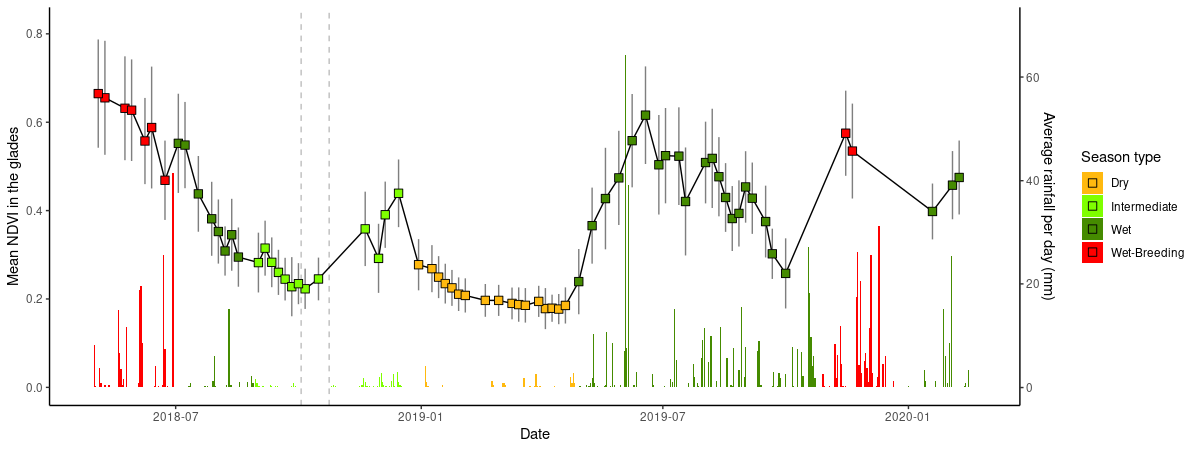
**

**Supplementary Figure 1.** Average NDVI on the glades, which are typical foraging areas for vulturine guineafowl, and total rainfall per day for each of the study periods. The dashed grey lines represent a period from which rainfall data wass missing.


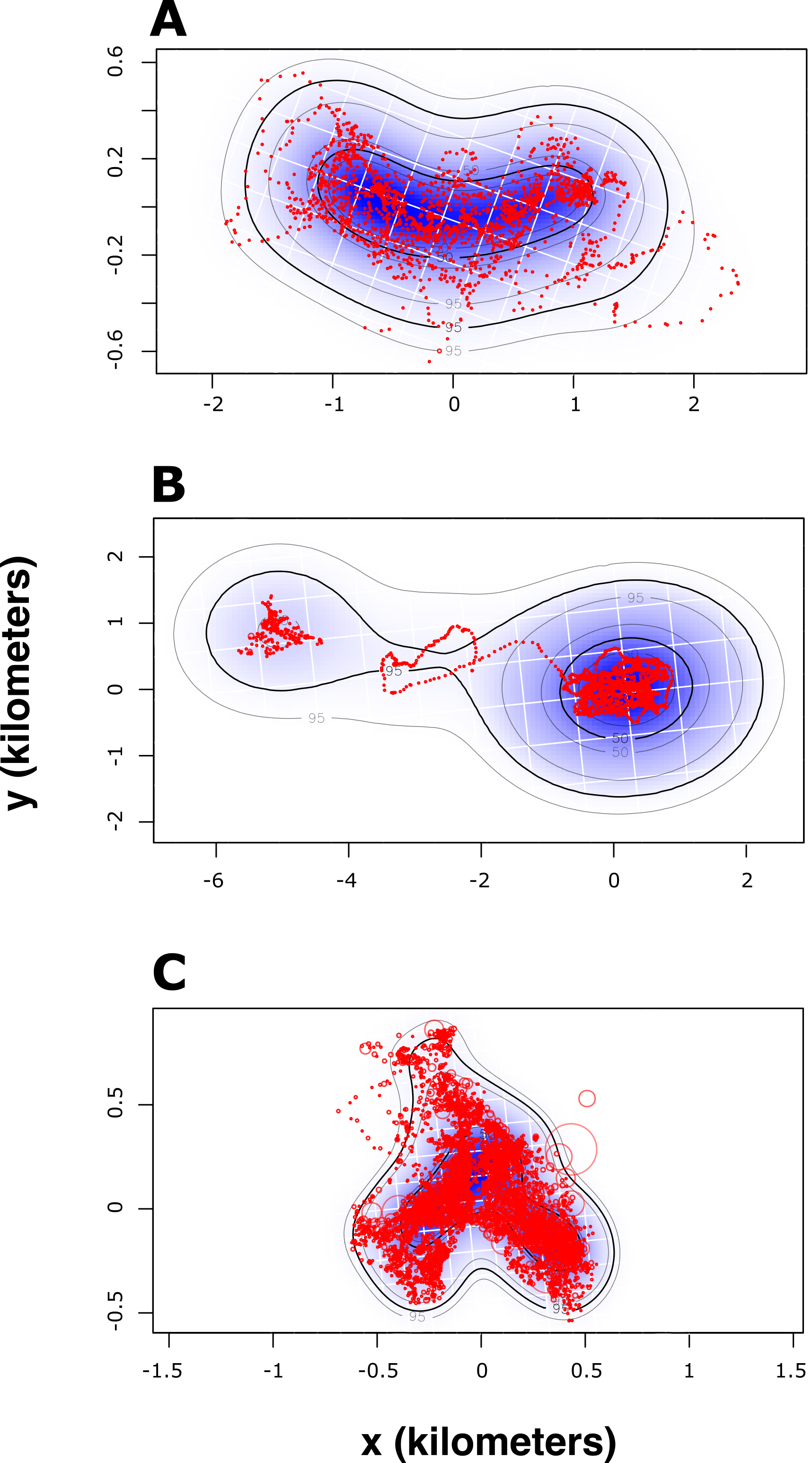


**Supplementary Figure 2. The 50% and 95% AKDEs, their confidence intervals and the distribution of GPS detections for one group in three seasons; (A)** intermediate season (light green polygon in Fig. 3, Season 4), **(B)** drought (yellow in Fig.3, Season 6) and **(C)** wet season that followed the drought (dark green, Season 7). Circles represent errors in the data.

**Supplementary References**

1. Copernicus Sentinel data. 2021.

2. SNAP - ESA Sentinel Application Platform v8.0.0.

3. Pettorelli N, Vik JO, Mysterud A, Gaillard J-M, Tucker CJ, Stenseth NC. Using the satellite-derived NDVI to assess ecological responses to environmental change. Trends Ecol Evol [Internet]. 2005;20:503–10. Available from: https://linkinghub.elsevier.com/retrieve/pii/S016953470500162X

4. Caylor KK, Gitonga J, Martins DJ. Mpala Research Centre Meteorological and Hydrological Dataset. Data File, Laikipia, Kenya: Mpala Research Centre.; 2020.
